# Efficient Use of Biological Data in the Web 3.0 Era by Applying Nonfungible Token Technology

**DOI:** 10.2196/46160

**Published:** 2024-05-28

**Authors:** Guanyi Wang, Chen Chen, Ziyu Jiang, Gang Li, Can Wu, Sheng Li

**Affiliations:** 1 Department of Urology Cancer Precision Diagnosis and Treatment and Translational Medicine Hubei Engineering Research Center Zhongnan Hospital, Wuhan University Wuhan China

**Keywords:** NFTs, biobanks, blockchains, health care, medical big data, sustainability, blockchain platform, platform, tracing, virtual, biomedical data, transformation, development, promoted

## Abstract

CryptoKitties, a trendy game on Ethereum that is an open-source public blockchain platform with a smart contract function, brought nonfungible tokens (NFTs) into the public eye in 2017. NFTs are popular because of their nonfungible properties and their unique and irreplaceable nature in the real world. The embryonic form of NFTs can be traced back to a P2P network protocol improved based on Bitcoin in 2012 that can realize decentralized digital asset transactions. NFTs have recently gained much attention and have shown an unprecedented explosive growth trend. Herein, the concept of digital asset NFTs is introduced into the medical and health field to conduct a subversive discussion on biobank operations. By converting biomedical data into NFTs, the collection and circulation of samples can be accelerated, and the transformation of resources can be promoted. In conclusion, the biobank can achieve sustainable development through “decentralization.”

## Dilemma of Sustainable Development of Biobanks

### Growth of Biobanks

Biobanks are biological application systems that are composed of physical samples and databases that centrally store various biological materials and their corresponding information for clinical treatments and life science research. Specific functions of biobank include standardized collection, processing, and storing of physical samples such as tissues, body fluids, and biological derivatives (eg, DNA and RNA), as well as related data storage such as pathological data, clinical diagnosis, treatment records, laboratory omics, ethical approval statements, and informed consent forms [1]. The rudimentary concept of a biobank was born in 1996 [2], and their numbers have increased yearly in the past 20 years with rapid developments in the biomedical industry. Around 636 biobanks opened in the United States in 2013 [3], and 325 institutions in the European-based Biobanking and BioMolecular resources Research Infrastructure were founded until 2018 [4]. However, most biobanks face problems with sustainable development.

### Current Dilemma

As biobanks are an essential link in the precision medicine industry, they require large-scale storage and sample and data management. Significant personnel and funding for equipment, technical services, biomedical consumables, and professional knowledge are necessary for early setup or maintenance. Moreover, biobanks undergo active or passive expansion as the storage sizes increase, which inevitably increases the need for funding [5]. However, as biobanks are not directly involved with production, they often act as an accessory to a specific research group or a collaborative department of public hospitals and have a fuzzy economic operation model [6]. In addition, international rules stipulate that stored human samples should not be used for commercial purposes [7], so establishing a reasonable operational model for biobanks and achieving sustainable development is critical and needs urgent discussion.

Currently, most biobanks in the world, such as the University of California San Francisco AIDS specimen bank, the Wales Cancer Biobank, the Australia Prostate Cancer Bioresource, and the Ontario Cancer Bank [8], recover costs by charging access fees from researchers. Despite this operational model, most biobanks cannot fully recover costs and rely solely on the support of government policies and donations. The Singapore biobank, established in 2002, eventually closed owing to a lack of finances and loss of public and government support [9]. Visibly, programs in which biobanks are developed solely by the government or with funding from a public agency are unrealistic. A path to achieving self-sufficiency is the only solution for long-term survival.

### Roots of the Dilemma

As biobanks form a bridge between clinical medicine and medical research, they must connect with patients who are willing to provide sample data resources and researchers of scientific institutes. Compared to researchers, patients have less initiative and are not as engaged in the entire industrial chain despite them being at the initial link of the chain as the sample and data source. One subjective reason is that patients usually do not fully understand and value the meaning of sample donation. The survey also evidenced that biobank participants are sometimes unaware of the potential risks and benefits of a project [10]; therefore, it may be challenging to convince them to donate.

Biobanks may alleviate funding dilemmas by charging samples and associated data from researchers. Nevertheless, this scheme does not completely resolve the financial burden and can cause other troubles. Relevant data show that sample use in biobanks is <10% [6]. The benefit from only a small number of samples and data may not be enough to afford the long-term management and storage expenses incurred by unused samples and data. In addition, it is difficult for biobanks to define the scope and quality of shared services when providing samples and data to researchers. For example, patient data, particularly those with chronic diseases, often dynamically increases with disease duration. Whereas the availability of biological medical data from biobanks cannot be a “one-time deal,” and determining how the access fee is charged is yet to be discussed. Moreover, security hazards such as data leakage during data sharing or informal access to data may affect the direct benefit of biobanks. It is noteworthy that even when biobanks take considerable economic benefits from operating on patient specimens and data, they can raise ethical and legal issues [11].

Besides ensuring adequate sample quantity, high-quality samples with complete data are prerequisites that guarantee subsequent scientific research output. Many larger biobanks are currently involved in converting physical samples into digital data besides storage management services. Thus, through comprehensive research and analysis of biological samples, genomics, proteomics, transcriptomics, and metabolomics data can be generated, which is convenient for storage and sharing. However, complete sample digitalization is unrealistic, and relying solely on biobanks is impractical. Failure in ensuring data integrity and comprehensiveness also affects subsequent sharing services that make it difficult for researchers to mine for valuable scientific information, in addition to other problems such as data standardization and sharing difficulties.

Throughout the entire industrial chain of translational medicine in biobanks, it is not difficult to find intrinsic motivation weak under its promising prospect. “Biobank” seems to exist merely as “storage” and does not reflect the actual connotation of “bank,” that is, to realize the distribution, value-added, and accelerated turnover of “resource wealth.” Perhaps, biobanks should be positioned not only as pipelines connecting upstream and downstream data but also as self-powered accelerators that vigorously drive research output while robustly accumulating data from clinical samples. Therefore, we introduce the concept of a nonfungible token (NFT) for digital assets that envision a new underlying model to address the existing issues.

## NFT of Biobank Sample Data

### What Is an NFT

**Figure 1:**
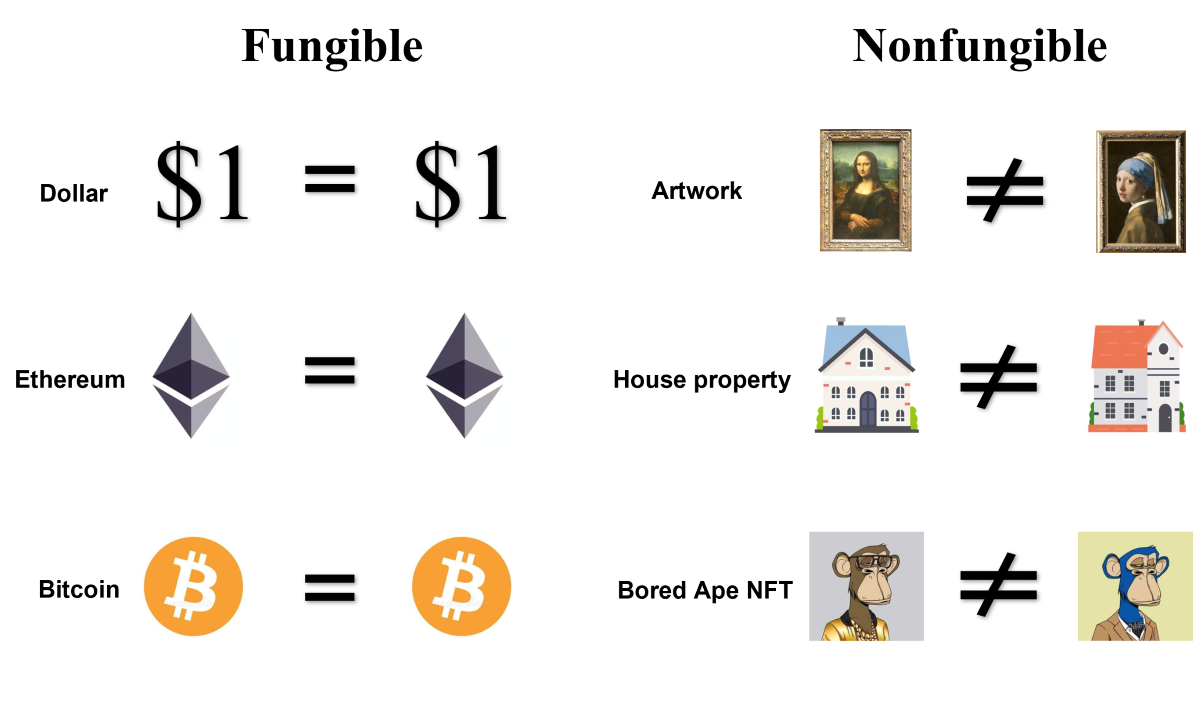
Differences between fungible assets and nonfungible assets. Fungible assets often have a fixed value and are concerned with their quantity rather than their specific attributes in any transaction. NFTs, like real estate, buildings, artworks, and gaming equipment, belong to the category of nonfungible assets, possessing uniqueness and nonreplaceability. NFT: nonfungible token.

In the real world, assets can generally be divided into fungible or nonfungible. Fungible assets such as stocks, game coins, and precious metals tend to have a fixed value that focuses on their quantity rather than their characteristics during any transaction. Unlike fungible assets, nonfungible assets such as houses, buildings, artworks, and game equipment are characterized by uniqueness and irreplaceability (Figure 1).

With the rise and rapid development of blockchain, digital assets have attracted wide attention from academia and industry. These are also classified into fungible and nonfungible assets corresponding to their value in the physical world. The most common fungible digital assets include digital currencies such as Bitcoins and Ethereum. However, nonfungible digital assets include NFTs such as game items and digital artworks [12]. NFTs make it possible to connect the real physical world with the digital network world and have become a core element at the center of an intelligent economy. NFT is a blockchain-based digital asset, which is verifiable, tradable, indivisible, irreplaceable, and unique. Ownership of an NFT can be transferred through intelligent contracts, and this transaction can be recorded by the blockchain; all transaction records can be viewed through any node. The characteristics of blockchain comprise open, transparent, traceable, anticounterfeit, and tamper-proof data that ensure transparency, tamper-proof, and antiduplication of the NFT transaction process [13].

### Feasibility of NFT for Biomedical Data

NFT has been successfully applied to many industries. However, it is mainly comprised of digital assets such as artwork, collectibles, video game items, and property rights. Bamakan et al [14] offered a layered conceptual NFT-based framework to create a decentralized market for patent storage and recording that facilitates changing the patent ownership and accelerates their commercialization. In addition, Dos Santos et al [15] proposed the use of NFTs and other tokens to realize transparency of supply chains and trace product supervision from the source and ensure food safety, enhance consumer trust, and realize sustainable agricultural development. With the maturity of blockchain technology and the popularization of its applications, transforming various digital assets and even physical assets into certifications will be a general trend due to the essential advantages of NFT in rights confirmation and transaction.

So far, NFT has not been deployed on a large scale in the field of biomedicine. Ng et al [16] systematically reviewed cutting-edge research on blockchain applications and other digital technologies in medicine and health care. They concluded a strong trend of digitization, such as the application of information technology in the public health response to COVID-19 [17], and blockchain-based patient-centric data management system for managing electronic health records in cancer nursing [18]. Digitalization of medical data will be a good start for NFT construction. NFT relies on a decentralized network that uses advanced encryption to verify the efficacy of transactions. Unlike cryptocurrencies that are seen as currency or digital assets, NFT is equivalent to a digital fingerprint of data or an authenticity certificate. It can hold many files that include transaction IDs and detailed history. Therefore, we can store a large number of biomedical and sample data through an NFT [19]. As envisaged by Skalidis et al [20], tagging and converting patient data (medical information, health status, examination results, and consent forms) into an NFT can enhance privacy and ensure data integrity and confidentiality in clinical practice and research.

### NFTs’ Potential Applications in the Biobanks

#### Data Security and Integrity

Biobanks are vital resources for medical research, containing extensive biological samples and related clinical data. The security and integrity of these data are crucial to ensure its credibility and availability. NFTs leverage blockchain technology to provide a decentralized method of data storage and management, effectively safeguarding data from unauthorized access and tampering. Each data transaction is recorded on the blockchain, ensuring data immutability, which helps maintain data integrity and protect patient privacy.

#### Data Traceability and Credibility

Data in biobanks often require tracing their sources and histories to ensure their credibility. NFTs, with the distributed nature and transparency of blockchain technology, make it easy to trace the origin and modification history of each data point. This is particularly helpful for verifying data accuracy and credibility, especially when validating the source and the quality of data is essential in medical research.

#### Data Standardization and Sharing

Due to varying standards in data collection, organization, and storage, there is a high degree of heterogeneity among biobanks. Undoubtedly, the lack of standardization leads to difficulties in sharing, which hinders subsequent research translation. By relying on NFTs’ standardization and universality, data storage could potentially break the barriers of nonstandardized sharing. Additionally, incidents of patient privacy breaches are continually emerging. The Georgia-based Emory Clinic experienced unauthorized data access in March 2017, resulting in the exposure of personal information for 79,930 individuals. The Alberta Health Service recently faced a privacy breach that leaked confidential health information for 12,848 individuals [11]. These security issues can be resolved through NFT’s blockchain-based sharing model.

#### Application of Smart Contracts

NFT-based smart contracts can programmatically execute contract conditions, providing a way for biobanks to automate the management of data and samples. Unlike other research activities, research using samples from biobanks has specific ethical and social problems owing to their long-term storage. So far, most biobanks have adopted the “broad consent” model, which is an agreement to use samples for current or future research within a specific framework without any subsequent contact with patients [21]. This provides researchers with enough flexibility to adapt to future research topics, but it also means that patients will not receive any feedback from the study. To some extent, “broad consent” cannot fully comply with the principle of “informed consent.” The World Medical Association Declaration of Helsinki in 1964 stipulated that every potential subject must be fully informed about any study-related information. Therefore, relying on the programmability of NFTs equipped with the corresponding service platform, we can achieve “dynamic consent” through smart contracts [22,23]. This approach places participants at the center of biobank governance, ensuring their data are only used with explicit consent. Patients can access “real-time” information about research projects and easily grant or revoke consent. This dynamic consent model increases participant control over the research process and enhances engagement while avoiding theoretical and legal loopholes in the “broad consent” model.

#### Decentralized Power Structure

**Figure 2:**
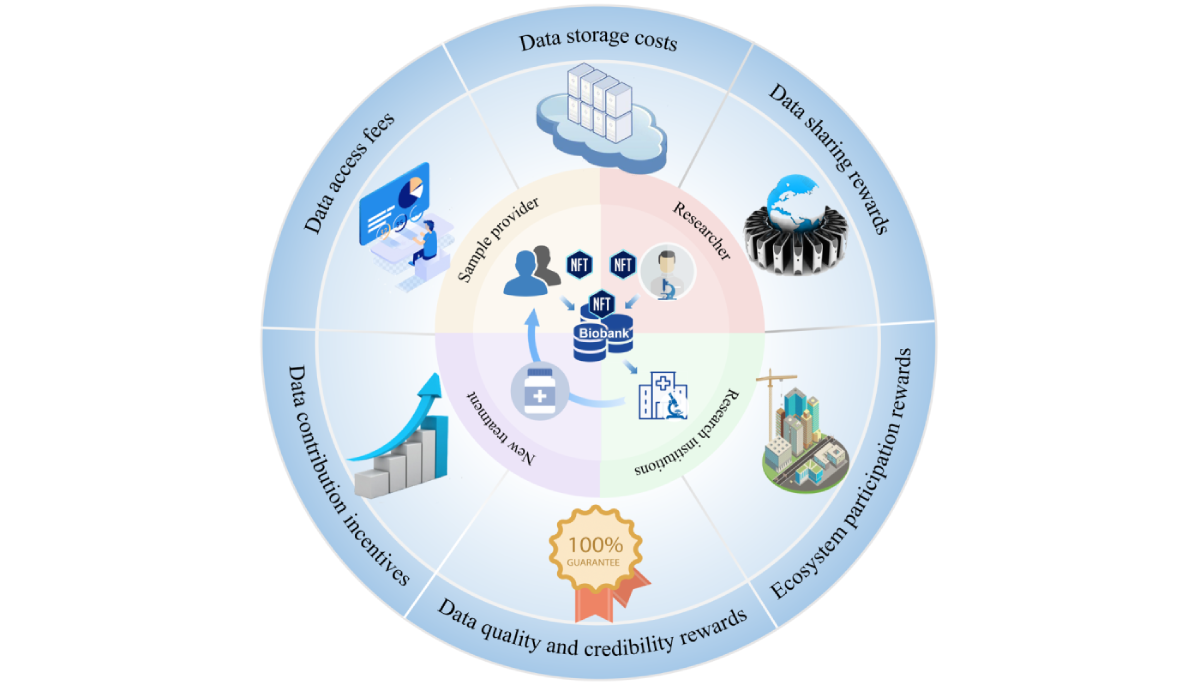
Achieving sustainable biobanks through NFTs. Clinical data from patients and research data from scientists are stored within the biobank, simultaneously generating 3 types of NFTs: the patient’s clinical data NFT, the researcher’s research data NFT, and the storage data NFT for the samples. The data within the biobank are subsequently mined by research institutions, ultimately resulting in research outputs such as new drugs or treatment protocols that benefit the patients themselves. Due to the inherent value of NFTs, there is an incentive for the storage of both clinical and research data. The storage data NFT for the samples can be considered as the self-generated value of the biobank, while the other 2 types of NFTs are anchored to it during transactions. Additionally, we envision incentive and economic models for the sustainable development of biobanks based on NFT transactions, including data contribution incentives, data access fees, data storage costs, data quality and credibility rewards, and data sharing rewards. NFT: nonfungible token.

“Decentralization” is a significant feature of organizations with blockchain applications in the Web 3.0 era, and its impact on biobanks is no exception. Traditional biobanks are typically managed and controlled by central institutions, which can lead to data centralization and potential misuse. Blockchain technology can achieve a decentralized power structure, transferring control of data to data owners, such as patients. This decentralization can increase data owner involvement and control while enhancing data availability. Patients often do not receive direct benefits from biological samples or data donations, and their potential benefit lies in the results or findings of scientific research projects in which they have participated. Patients can benefit earlier from the industrial chain after their data are converted into NFTs. They do not need to fully understand the value of samples in scientific research projects, and donation can shift from a seemingly moral act of “giving” to a direct “benefit exchange” behavior. Depending on the data source, NFTs include clinical data NFTs, owned by patients, experimental data NFTs generated by researchers, and sample information NFTs generated during the storage process. Rights and power relations between research institutions, individual researchers, and participants can be explicitly defined through NFT ownership. This resolves the complex ethical issue of who owns and controls biobank data. As indicated by Kostick-Quenet et al [24], NFTs or NFT-like frameworks can incentivize a more democratic, transparent, and efficient health information exchange. In our vision, biobanks will decentralize their rights and responsibilities to both upstream and downstream data, achieving a beneficial situation (Figure 2).

## Achieving Sustainable Biobanks Through NFTs

### Overview

NFT technology can also improve the economic model of biobanks. Biobanks should be able to operate much like banks and sustain their operations by earning “interest,” thus reducing their reliance on government funding. To achieve this, we propose the following incentive and economic models.

### Data Contribution Incentives

Biobanks can motivate individuals or research institutions to contribute their biomedical data to the repository through reward mechanisms. When individuals or institutions convert their data into NFTs and store them on the blockchain, they can earn cryptocurrency or other rewards. This incentive model encourages more data contributions, increasing the quantity and diversity of data in the repository.

### Data Access Fees

Researchers or health care institutions seeking access to the repository’s data can pay data access fees. These fees can be determined based on the value and scarcity of the data and can be automatically processed by smart contracts. Some of the data access fees can be allocated to the maintenance and operation of the repository, ensuring its sustainability.

### Data Storage Costs

Individuals or institutions storing their data in the repository may need to pay storage fees. This encourages data owners to update and manage their data promptly, avoiding wastage of storage space. Storage fees can also serve as a source of income for the biobank, supporting its operations.

### Data Quality and Credibility Rewards

Data quality and credibility are crucial for medical research. The repository can establish reward mechanisms to incentivize data contributors to maintain high-quality, accurate, and complete data. These rewards can be provided in the form of NFTs or cryptocurrencies to encourage data contributors to provide the best data.

### Ecosystem Participation Rewards

Parties participating in the biobank ecosystem, including researchers, health care institutions, developers, and maintainers, can earn ecosystem participation rewards. These rewards can be determined based on their contributions and level of involvement in the ecosystem. This helps build an actively engaged community, driving the growth of the repository.

### Data Sharing Rewards

When data are used multiple times or shared across various research projects, data contributors can earn rewards. This incentivizes the reuse and sharing of data, increasing its value.

These incentive and economic models can be automatically executed on the blockchain through smart contracts, ensuring fairness and transparency. They encourage best practices in data contribution, management, access, and quality, thereby increasing the value and sustainability of biobanks. Moreover, these models contribute to the establishment of a shared and collaborative medical research ecosystem, driving advancements in medical research.

## NFT-Based Biomedical Data Framework

### Overview

**Figure 3:**
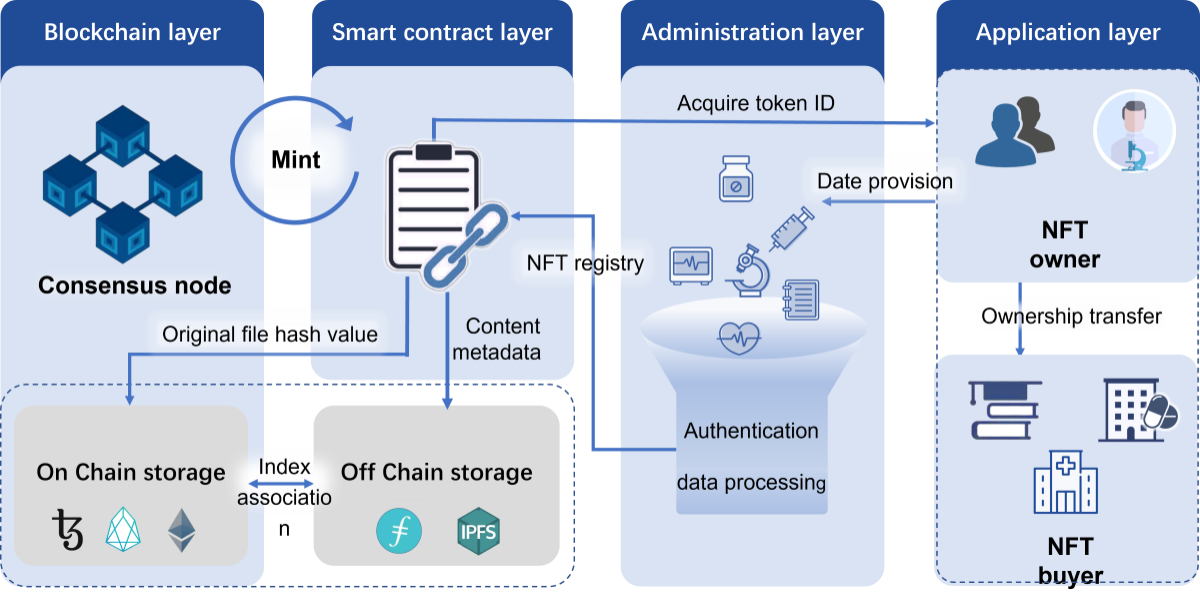
Proposed architecture for presenting NFT-based biomedical data. A basic framework of biomedical data based on an NFT, which includes 4 primary levels: application layer, management layer, smart contract layer, and blockchain network layer. Creating NFTs for biomedical data and leveraging the properties of NFTs can indeed be beneficial for improving the efficiency of data conversion and sharing within biobanks. NFT: nonfungible token.

Based on the above assumption, we propose a basic framework of biomedical data based on an NFT, which includes 4 primary levels: application layer, management layer, smart contract layer, and blockchain network layer (Figure 3). The specific structure and implementation of the entire framework are as follows.

### Application Layer

By interacting with decenteralized applications, the biomedical data platform allows data providers such as patients and researchers to input or import data via interfaces. After the data are marked as an NFT, its ownership will be transferred to the data demander (schools, hospitals, pharmaceutical enterprises, and other scientific research institutions). Ownership will be eventually tracked through the blockchain.

### Management Level

This will implement user registration and authentication management and use the data processing module to desensitize, clean, and fuse the data to ensure data efficacy and security. Implementation of the interface operation will be done with the NFT smart contract.

### Smart Contract Layer

Smart contracts generated related to biomedical information as programs running on the blockchain can receive and send transactions. However, they must comply with NFT protocols like Ethereum’s erc721 protocol [25]. After generating a smart contract, the user will obtain a token ID as the NFT ownership certificate. The hash value of the original biomedical information file will be uploaded to the blockchain network for broadcasting, and the metadata of the original file will be stored in the chain [26,27]. The so-called “Mint” process records the ID of an NFT and the address of its owner on the blockchain.

### Blockchain Network Layer

Blockchain systems can be mainly divided into public and private blockchains based on their consensus mechanisms. In public blockchains, any node can participate in the point-to-point network, and it is completely decentralized. In a private blockchain, the read permission can be opened to the public or it can be arbitrarily restricted based on the situation. In addition to these, there is also a semidecentralized alliance chain whose consensus process may be controlled by some designated nodes [28,29]. Public chains with good credit can be selected, such as Ethereum, EOS, Flow, Tezos, and so forth.

### Off-Chain Storage

Biomedical data are rich in content, and the chain of original files tends to put tremendous pressure on the blockchain network. Thus, the chain of original files is not realistic at present, and off-chain storage can solve the network storage pressure, network pressure, and high maintenance costs [30]. Developers use a function (token uniform resource identifier) to let the app know the location of the metadata of a specific item and establish on-chain and off-chain indexes to realize the association [25,31]. Common off-chain external storage platforms include interPlanetary File System, Filecoin, and Pinata.

Based on the above framework, NFT technology presents potential solutions for the biobank field, enhancing data security, credibility, and management efficiency while also increasing data owners’ participation and control. These technologies hold promise for significant improvements in biobank operations and medical research, providing better data management and use for both patients and researchers.

## Future and Development

We are amidst the tremendous transformation of the Web 2.0 era into the Web 3.0 era. This requires joint exploration of multiple technologies, including blockchain, artificial intelligence, and the internet of things. Ultimately, we will embrace a relatively decentralized, automated, and intelligent new era of the internet based on the premise that personal digital identity, digital assets, and data return would be entirely secure for individuals. In addition to changes in the web-based network, continuous interaction between the digital and the real world and developing future technologies will bring new subversive actions to the social systems and institutions in the physical world. In terms of ethical issues, web-based world ethics is usually an extension or reflection of real ethics. The interaction of virtual reality makes personal data more widely collected and shared, and there are more potential unknown risks. In this situation, protecting personal privacy and data security has become crucial. Therefore, it is necessary to establish emerging ethical and legal norms to balance the relationship between technological development and individual rights. Ethics committees or review bodies at the level of hospitals and research institutions, as well as higher level supervisory bodies, should focus more on the authenticity, security, and traceability of data and ensure the transparency and effectiveness of ethical review and supervision agencies. In addition, with the decentralized development model of the Web 3.0 era, after desensitizing relevant data, the supervision and management rights of such relevant institutions can even be delegated to every data participant.

Currently, medical institutions in many countries offer enhanced views of precision 3D imaging to physicians using digitally assisted procedures during the treatment, such as applying Microsoft HoloLens 2 hybrid reality technology in high-finesse surgical procedures [32,33]. Similarly, in the future, with the development of XR (virtual reality, augmented reality, mixed reality) technology, 6G high-speed network transmission, and cloud computing, we could eventually meet all requirements of the “all-round true experience” of the metaverse and can provide a better treatment experience and visits to patients.

In the Web 3.0 era, “all-round true experience” will break through the barriers of time and space, making medical resources more evenly shared. Finally, the importance of individual physician’s autonomy and data exchange platform will increase. Perhaps biobanks could be a foundational platform for the convenient sharing of all health care data in the Web 3.0 era. In addition to clinical and experimental data, remote monitoring of health and epidemiology data generated by wearable devices, implanted microchips, and the internet of things will be incorporated into storage and management [34].

We also hypothesize that an open-source, public blockchain platform with an intelligent contract function could also be developed in public health, which could breach the barrier of data silos among various hospitals, medical research institutes, and drug development enterprises. Thus, we can create a cosmopolitan project using human health data and build a new world for revolutionary data management.

## Abbreviations

NFT: nonfungible token
